# Effect of different storage solutions on oxidative stress in human saphenous vein grafts

**DOI:** 10.1186/s13019-022-01752-7

**Published:** 2022-01-16

**Authors:** Ilker Tekin, Meltem Demir, Sebahat Özdem

**Affiliations:** 1 Department of Cardiovascular Surgery, Manavgat Government Hospital, Manavgat, Turkey; 2 Department of Cardiovascular Surgery, Faculty of Medicine, Bahçeşehir University, İstanbul, Turkey; 3 Department of Biochemistry, Medicalpark Hospital Complex, Antalya, Turkey; 4 Faculty of Health Science, Bilim University, Antalya, Turkey; 5 Department of Medical Biochemistry, Faculty of Medicine, Akdeniz University, Antalya, Turkey

**Keywords:** Vein graft failure, Coronary artery bypass graft, Storage solution, Endothelial damage inhibitor

## Abstract

**Background:**

Ischemia–reperfusion injury of saphenous vein grafts (SVG) during coronary artery bypass grafting surgery negatively impacts endothelial integrity and functionality and is associated with vein graft failure. The aim of this study was to evaluate the level of oxidative stress in human SVG segments following ischemic storage in three intraoperative graft storage solutions: saline (S), autologous heparinized blood (HB) and DuraGraft (DG).

**Methods:**

3 mm tissue rings derived from surplus SVG segments from 50 patients were stored at room temperature for 30 min in DG, S or HB. Total oxidative status (TOS) and total antioxidant status (TAS) levels were determined from which the oxidative stress index (OSI: TOS/TAS ratio) was calculated. A *p*-value < 0.017 was considered significant implementing a Bonferroni correction.

**Results:**

TOS values were significantly lower for DG stored samples in comparison to both S and HB; there was no difference between S and HB (DG: 32.6 ± 1.8, S: 39.6 ± 2.8 and HB: 40.6 ± 2.4 µmol H_2_O_2_ eqv.; DG vs. S and DG vs. HB *p* < 0.0001, S vs. HB *p* = 0.047). TAS was higher for both DG and HB in comparison to S (DG: 8.9 ± 0.9, S: 6.9 ± 1.0 and HB: 8.6 ± 0.9 mmol Trolox eqv.; DG vs S *p* < 0.0001, DG vs. HB p = 0.263, S vs. HB *p* < 0.0001). OSI differed between all groups with the lowest value for DG (DG: 3.7 ± 0.2, S: 5.8 ± 0.4 and HB: 4.7 ± 0.2 µmol H_2_O_2_ eqv./mmol Trolox eqv.; all *p* < 0.0001).

**Conclusions:**

Saphenous veins grafts stored in DuraGraft had a lower oxidative level, higher antioxidant level and a lower oxidative stress index in comparison to saphenous vein grafts stored in saline or heparinized blood.

*ClinicalTrials.gov Identifier* NCT02922088.

## Background

Coronary artery bypass graft (CABG) surgery remains the treatment of choice for patients with multivessel and/or left main disease with the saphenous vein graft (SVG) as most frequently used conduit [1, 2]. However, the durability and patency of SVGs are significantly compromised by vein graft disease (VGD). Vein graft failure (VGF), the end-stage of VGD, is associated with recurrent angina, myocardial infarction, repeat revascularization and death [3, 4]. Studies have reported incidences of VGF at graft level of 15–29% at 1 year to up to 50–60% at 10 year [5, 6].

VGD is initiated by damage that occurs to the graft intraoperatively and in particular damage to the graft’s endothelial layer. While graft injury can occur from various physical stresses during harvest and handling (e.g. surgical trauma, graft over-pressurization during flushing), the primary mediator of intraoperative graft damage is ischemia–reperfusion injury (IRI) that occurs between graft harvesting and anastomosis [7].

Therefore, sufficient graft preservation that limits intra-operative IRI is a major determinant to maintain graft patency [8]. Preservation solutions are designed to be biocompatible with human tissue and contain components that directly or indirectly interfere with the principal mechanism of IRI, i.e. oxidative damage [9]. Since oxidative damage is mediated through the release of reactive oxygen species (ROS) and other oxidants from endothelial and other cells in the ischemic tissue, preservation solutions must have sufficient antioxidant activity to neutralize ROS in order to prevent oxidative damage.

To date, vascular grafts are intra-operative temporarily stored in saline- or blood-based solutions despite being not pH neutral, not biocompatible and not protective against IRI, while clinical studies have demonstrated their negative impact on 12-month graft failure rates [10]. pH buffered solutions, although better, only modestly reduced the 12-month failure rates [9, 11, 12]. To this end, DuraGraft (DG), an intraoperative endothelial damage inhibitor, has been developed for graft preservation. DG is based on a physiological salt solution containing a synergistic cocktail of potent antioxidants to prevent IRI. The aim of this study was to compare the overall oxidative stress levels in SVGs after storage in DG, saline (S) and heparinized blood (HB). Evaluation was performed by measuring total oxidant status, total antioxidant status and by calculating the oxidative stress index.

## Methods

### Patients

SVGs were harvested from a total of 50 patients undergoing isolated or combined CABG using the standard hospital protocol. Graft harvesting was performed by a single operator (IT) and biochemical analysis were performed by a single analysist (MD). Patients were selected from the European Multicenter Registry to Assess Outcomes in CABG Patients: Treatment of Vascular Conduits With DuraGraft (ClinicalTrials.gov Identifier: NCT02922088), but this biochemistry sub-study was physician initiated and conducted independent from the sponsor [13]. It was approved by the hospital ethical committee and all patients provided written informed consent.

### Graft storage solutions

DuraGraft™ (Marizyme, Jupiter, Florida, USA) is an ionically and pH-balanced physiological salt solution containing the antioxidants L- glutathione and L-ascorbic acid as well as L-arginine and glucose. DuraGraft is supplied as a two-container system; the two solutions are mixed at point-of use to create the preservation solutions used in the operating theatre [14]. Heparin is added before use based on the standard practice of each center. The other storage solutions were saline (S) (0.9% sodium chloride) and autologous heparinized blood (HB).

### Graft sampling and biochemical analysis

The surplus of SVG that was not used for CABG was used for the purpose of this study. From each surplus SVG segment, rings of 3 mm were cut and stored in the respective storage solutions at regular operating room temperature for 30 min. Subsequently, the samples were immediately stored at −80 °C. After defrosting, supernatants were generated by homogenizing the samples in a glass homogenizer with 300 µl phosphate buffered saline at pH 7.4. The homogenates were centrifuged at 10,000 × *g* for 10 min at 4 °C after which the supernatants were removed for analysis. Results were expressed as units per gram of protein in the supernatant.

Supernatant total oxidative status (TOS) and total antioxidant status (TAS) levels were determined with commercially available kits (Rel Assay Diagnostics, Gaziantep, Turkey) and Oxidative Stress Index (OSI) values were calculated as the ratio of TOS to TAS. TOS outcomes represent the level of oxidant molecules present in the sample and indicate oxidative stress levels. TAS outcomes represent the overall antioxidant status, i.e. the ability to neutralize ROS and therefore prevent cellular damage caused by ROS. TOS levels were determined spectrophotometrically using a method described by Erel, and the results were expressed in terms of micromolar hydrogen peroxide equivalent per liter (µmol H2O2 eqv.) [15]. TAS levels were determined using the fully automated spectrophotometric method developed by Erel [15, 16]. The results were expressed as milimolar Trolox equivalent per gram (mmol Trolox eqv.).

### Statistical analysis

Categorical variables are presented as frequencies and percentages. Continuous variables are displayed as means ± standard deviation. The Shapiro–Wilk test of normality was used to test for normal distribution of the biochemical test results among treatment groups (DG, S, HB). Differences between treatment groups for the biochemical tests were evaluated by paired t-test. If the assumption of normality was not met, the nonparametric Wilcoxson rank sum test was used to test differences between treatment groups. To account for multiple comparisons, a Bonferroni correction was applied and a *p*-value of 0.017 was considered significant. All statistical analysis was performed with SAS software, version 9.4 (SAS institute, Cary, NC, USA).

## Results

**Fig. 1:**
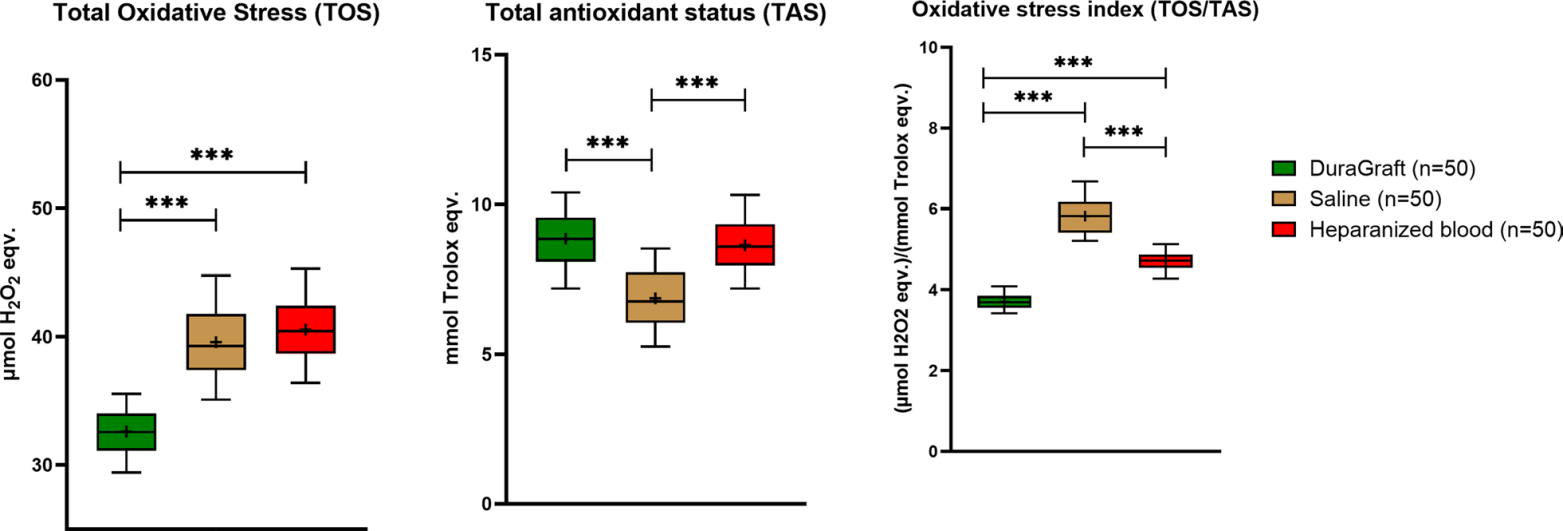
Box and whisker plot representing the biochemical outcomes after storage in DuraGraft, saline and heparinized autologous blood with the median, mean (+), 25th and 75th percentiles, min and max values. ***Indicates a *p*-value < 0.0001

SVG samples were obtained from 50 patients undergoing isolated or combined CABG and valve surgery and either stored in DG (n = 50), S (n = 50) or HB (n = 50). The majority of patient were male (84%). The prevalence in patient of diabetes mellitus, hypertension and dyslipidemia was 48%, 74% and 36% of the patients, respectively (Table 1). Biochemical analysis was performed for all SVG samples from all patients after storage in the respective solutions (Table 2 and Fig. 1). All significant differences were at a *p*-values < 0.0001. TOS values were similar for the S and HB group (39.6 ± 2.8 and 40.6 ± 2.4 µmol H_2_O_2_ eqv., *p* = 0.047), whereas the TOS values were significantly lower in DG treated SVG (32.6 ± 1.8 µmol H_2_O_2_ eqv.) compared to both HB and S. TAS was significantly lower in SVG segments stored in S (6.9 ± 1.0 mmol Trolox eqv.) compared to DG (8.9 ± 0.9 mmol Trolox eqv.) and HB (8.6 ± 0.9 mmol Trolox eqv.); no difference was observed between DG and HB (p = 0.263). The calculated OSI values were the lowest in the DG group (3.7 ± 0.2 µmol H_2_O_2_ eqv.)/mmol Trolox eqv.) in comparison to both S (5.8 ± 0.4 µmol H_2_O_2_ eqv.)/mmol Trolox eqv.) and HB (4.7 ± 0.2 µmol H_2_O_2_ eqv.)/mmol Trolox eqv.).

**Table 1:** Patient baseline and procedural characteristics

Characteristics	N = 50
Age, y, mean ± sd	64.5 ± 8.2
Age range, y	47–82
Male	42 (84.0%)
Height, cm, mean ± sd	168.2 ± 6.5
Weight, kg, mean ± sd	79.5 ± 14.0
BMI, kg/m^2^, mean ± sd	28.8 ± 4.3
Current smoker	24 (48.0%)
Diabetes mellitus	24 (48.0%)
Hypertension	37 (74.0%)
Dyslipidemia	18 (36.0%)
Renal failure	0 (0.0%)
Peripheral artery disease	5 (10.0%)
Number of distal anastomosis	
1	1 (2.0%)
2	2 (4.0%)
3	16 (32.0%)
4	24 (48.0%)
5	7 (14.0%)

**Table 2:** Biochemical outcomes after storage of saphenous vein grafts in DuraGraft, saline and heparinized autologous blood. A *p*-value < 0.017 was considered significant implementing a Bonferroni correction

	DuraGraft (DG) N = 50	Saline (S) N = 50	Heparinized blood (HB) N = 50	p-value DG vs S	p-value DG vs HB	p-value S vs HB
Total oxidative stress [TOS, µmol H_2_O_2_ eqv.]	32.6 ± 1.8	39.6 ± 2.8	40.6 ± 2.4	< 0.0001	< 0.0001	0.047
Total antioxidant status [TAS, mmol Trolox eqv.]	8.9 ± 0.9	6.9 ± 1.0	8.6 ± 0.9	< 0.0001	0.263	< 0.0001
Oxidative stress index [TOS (µmol H_2_O_2_ eqv.)/TAS (mmol Trolox eqv.)]	3.7 ± 0.2	5.8 ± 0.4	4.7 ± 0.2	< 0.0001	< 0.0001	< 0.0001

## Discussion

Rates of VGF and associated clinical events post-CABG surgery remain high despite intra-operative measures to prevent VGF including surgical techniques such as avoiding extensive handling during SVG harvesting, selecting the optimal site for the distal anastomosis to ensure a good run-off area, avoiding kinking and flattening of the graft and the no-touch harvesting technique [17]. These measures seek to preserve the integrity, functionality and viability of the endothelial layer, and to reduce the occurrence of early graft thrombosis and its clinical sequalae. Despite this, ischemic injury and associated oxidative damage have been identified as the primary driver of intraoperative endothelial injury that leads to VGD via an IRI mechanism.

IRI is initiated during ischemic episodes through damage caused by oxidative stress [8, 18]. Oxidative damage is mediated by the release of ROS from endothelial and other cells in the ischemic tissue and results in chemical modification of cellular and extracellular components. The net result is the loss of normal cell and matrix components leading to dead, non-functional, structurally perturbed and dysfunctional tissue, cells and matrix. Subsequent reperfusion of the ischemic organ does not restore normality but instead exacerbates damage incurred during ischemia [8, 18]. Similar to allotransplantation, prevention of IRI by storage of the SVG in a biocompatible medium that reduces ischemic injury is key to providing best patient outcomes following grafting [9].

The current study investigated oxidative stress indices in SVG samples stored in DG, an endothelial damage inhibitor designed for intra-operative graft preservation, versus samples stored in the standard of care solutions, S and HB. The main study finding is that SVGs stored in DG exhibit a statistically significant lower OSI compared to SVGs stored in either S or HB. Oxidative stress in the SVGs reflects a higher level of ROS and other oxidants compared to the levels of available antioxidants. As oxidant levels increasingly exceed the system’s antioxidant capacity, OSI increases and so does oxidative damage to cellular components.

The lower OSI in DG stored SVGs is the mathematical result of both lower TOS levels and higher TAS levels compared to S and HB. The antioxidants in DG, L-glutathione and L-ascorbic acid are known to inactivate oxidants which likely explains the lower TOS levels. The higher TAS levels in DG stored SVGs indicate that there is a higher reserve of antioxidants provided by unused L-glutathione and L-ascorbic acid molecules. Once L-glutathione or L-ascorbic acid inactivate an oxidant molecule, it becomes inactive itself and levels of antioxidants will decline. Overall, the lower OSI in DG stored SVG compared to grafts stored in S or HB is predicted to better protect SVGs from ischemic damage during ex-vivo storage and potentially to mitigate VGF.

The importance of the effect of storage solutions on clinical outcomes was investigated in a subgroup analysis of the PREVENT IV study [10]. Patients whose grafts were stored in a buffered solution had a lower VGF rate and a trend toward better long-term clinical outcomes compared to patients whose grafts were stored in unbuffered saline- or blood-based solutions.

The current findings are consistent with earlier studies conducted with DG. In an in-vitro and ex-vivo study that compared DG to S, human SVG and isolated pig mammary vein segments were flushed and submerged in DG and S for prespecified times [19]. Loss of human SVG cell viability was observed as early as 15 min post-exposure to S, whereas viability was maintained up to 5 h exposure to DG. Histological analyses of the pig mammary veins stored in S for 60 min demonstrated endothelial and other cell damage in contrast to DG stored veins that did not show evidence of damage or reactivity. In a recently published study, human SVG and radial artery segments were preserved in DG and heparinized lactated Ringer’s solution for 60 min [20]. A decreased ROS production and balanced conditions between oxidative and antioxidant values were observed after storage in DG. Furthermore, DG inhibited progressive neointimal formation by downregulation of transforming growth factor beta (TGFβ) induced vascular endothelial growth factor (VEGF) cellular over-proliferation. The authors conclude that DG treatment of radial artery grafts may significantly reduce post-grafting re-oxygenation reaction and may have the potential to reduce the occurrence of radial artery graft disease and failure in patients undergoing CABG.

In a human clinical study, the effect of DG on SVG early anatomical changes associated with VGD was assessed using multidetector computed tomography angiography at 1, 3, and 12 months post-CABG [14]. Within each patient, two SVGs were randomized to either DG or S to exclude differences in patient characteristics as a confounding factor. DG was found to have a favorable effect on early anatomical markers of VGD such as a smaller SVG wall thickness at 12 months, particularly in the proximal segment of the graft where early disease has been shown to be most frequently manifest [21]. To further assess the performance of DG, a 3000 patients registry including patients that underwent isolated CABG as well as combined CABG and valve surgery has been initiated [13]. Enrollment has been completed end of 2019, follow-up is ongoing and the first results are eagerly awaited.

## Strengths and limitations of the study

This study was conducted at a single center, the SVGs were harvested by the same surgeon and the biochemical analysis were performed by the same analyst. This importantly reduces the variation in SVG harvesting technique and analysis methodology. Two regularly used storage solutions and one solution specifically developed for graft storage have been tested. Moreover, samples from a large number of patients representative of patients undergoing CABG have been studied. These design elements illustrate the robust design of the study and substantiate the validity of the data. A limitation is that no heparin was added to S conform the center’s practice, unlike the addition of heparin to DG and HB. It should further be acknowledged that the pathophysiological and clinical relevance of the findings need further research.

## Conclusion

In conclusion, SVGs intra-operatively stored in DuraGraft showed a lower oxidative level, higher antioxidant level and a lower oxidative stress index in comparison to saphenous vein grafts stored in saline or heparinized blood. This could have implications for the prevention of vein graft disease and subsequent failure and warrant further investigation. While well designed studies are needed to confirm these hypothesis generating findings, the use of dedicated graft preservation solutions with anti-oxidant characteristics are predicted to increase saphenous vein graft patency rates thereby improving long-term clinical outcomes following CABG surgery.

## Data Availability

Authors confirm that all relevant data are included in the article.

## Abbreviations

CABG: Coronary artery bypass grafting
HB: Heparinized blood
IRI: Ischemia-reperfusion injury
OSI: Oxidative stress index
S: Saline (0.9% saline)
ROS: Reactive oxygen species
SVG: Saphenous vein graft
TAS: Total antioxidant status
TOS: Total oxidative stress
VGD: Vein graft disease
VGF: Vein graft failure

## References

[1] Sousa-Uva M, Neumann FJ, Ahlsson A, Alfonso F, Banning AP, Benedetto U, Byrne RA, Collet JP, Falk V, Head SJ, Jüni P, Kastrati A, Koller A, Kristensen SD, Niebauer J, Richter DJ, Seferovic PM, Sibbing D, Stefanini GG, Windecker S, Yadav R, Zembala MO; ESC Scientific Document Group. 2018 ESC/EACTS Guidelines on myocardial revascularization. Eur J Cardiothorac Surg. 2019 1;55:4–90

[2] Caliskan E, de Souza DR, Böning A, Liakopoulos OJ, Choi YH, Pepper J, Gibson CM, Perrault LP, Wolf RK, Kim KB, Emmert MY (2020). Saphenous vein grafts in contemporary coronary artery bypass graft surgery. Nat Rev Cardiol.

[3] Halabi AR, Alexander JH, Shaw LK, Lorenz TJ, Liao L, Kong DF, Milano CA, Harrington RA, Smith PK (2005). Relation of early saphenous vein graft failure to outcomes following coronary artery bypass surgery. Am J Cardiol.

[4] Yau JM, Alexander JH, Hafley G, Mahaffey KW, Mack MJ, Kouchoukos N, Goyal A, Peterson ED, Gibson CM, Califf RM, Harrington RA, Ferguson TB; PREVENT IV Investigators. Impact of perioperative myocardial infarction on angiographic and clinical outcomes following coronary artery bypass grafting (from PRoject of Ex-vivo Vein graft ENgineering via Transfection [PREVENT] IV). Am J Cardiol. 2008;102:546–51 10.1016/j.amjcard.2008.04.069 18721510

[5] Fitzgibbon GM, Kafka HP, Leach AJ, Keon WJ, Hooper GD, Burton JR (1996). Coronary bypass graft fate and patient outcome: angiographic follow-up of 5,065 grafts related to survival and reoperation in 1,388 patients during 25 years. J Am Coll Cardiol.

[6] Deb S, Cohen EA, Singh SK, Une D, Laupacis A, Fremes SE; RAPS Investigators. Radial artery and saphenous vein patency more than 5 years after coronary artery bypass surgery: results from RAPS (Radial Artery Patency Study). J Am Coll Cardiol. 2012;60:28–35. 10.1016/j.jacc.2012.03.037 22742399

[7] Shuhaiber JH, Evans AN, Massad MG, Geha AS (2002). Mechanisms and future directions for prevention of vein graft failure in coronary bypass surgery. Eur J Cardiothorac Surg.

[8] Osgood MJ, Hocking KM, Voskresensky IV, Li FD, Komalavilas P, Cheung-Flynn J, Brophy CM (2014). Surgical vein graft preparation promotes cellular dysfunction, oxidative stress, and intimal hyperplasia in human saphenous vein. J Vasc Surg.

[9] Latchana N, Peck JR, Whitson BA, Henry ML, Elkhammas EA, Black SM (2015). Preservation solutions used during abdominal transplantation: current status and outcomes. World J Transplant.

[10] Harskamp RE, Alexander JH, Schulte PJ, Brophy CM, Mack MJ, Peterson ED, Williams JB, Gibson CM, Califf RM, Kouchoukos NT, Harrington RA, Ferguson TB, Lopes RD (2014). Vein graft preservation solutions, patency, and outcomes after coronary artery bypass graft surgery: follow-up from the PREVENT IV randomized clinical trial. JAMA Surg.

[11] Wise ES, Hocking KM, Eagle S, Absi T, Komalavilas P, Cheung-Flynn J, Brophy CM (2015). Preservation solution impacts physiologic function and cellular viability of human saphenous vein graft. Surgery.

[12] Williams JB, Harskamp RE, Bose S, Lawson JH, Alexander JH, Smith PK, Lopes RD (2015). The preservation and handling of vein grafts in current surgical practice: findings of a survey among cardiovascular surgeons of top-ranked US Hospitals. JAMA Surg.

[13] Caliskan E, Sandner S, Misfeld M, Aramendi J, Salzberg SP, Choi YH, Satishchandran V, Iyer G, Perrault LP, Böning A, Emmert MY (2019). A novel endothelial damage inhibitor for the treatment of vascular conduits in coronary artery bypass grafting: protocol and rationale for the European, multicentre, prospective, observational DuraGraft registry. J Cardiothorac Surg.

[14] Perrault LP, Carrier M, Voisine P, Olsen PS, Noiseux N, Jeanmart H, Cardemartiri F, Veerasingam D, Brown C, Guertin MC, Satishchandran V, Goeken T, Emmert MY (2021). Sequential multidetector computed tomography assessments after venous graft treatment solution in coronary artery bypass grafting. J Thorac Cardiovasc Surg.

[15] Erel O (2004). A novel automated method to measure total antioxidant response against potent free radical reactions. Clin Biochem.

[16] Erel O (2005). A new automated colorimetric method for measuring total oxidant status. Clin Biochem.

[17] Souza D (1996). A new no-touch preparation technique. Technical notes. Scand J Thorac Cardiovasc Surg.

[18] Granger DN, Kvietys PR (2015). Reperfusion injury and reactive oxygen species: the evolution of a concept. Redox Biol.

[19] Pachuk CJ, Rushton-Smith SK, Emmert MY (2019). Intraoperative storage of saphenous vein grafts in coronary artery bypass grafting. Expert Rev Med Devices.

[20] Aschacher T, Baranyi U, Aschacher O, Eichmair E, Messner B, Zimpfer D, Moayedifar R, Laufer G, Emmert MY, Sandner SE (2021). A novel endothelial damage inhibitor reduces oxidative stress and improves cellular integrity in radial artery grafts for coronary artery bypass. Front Cardiovasc Med.

[21] Berkowitz HD, Fox AD, Deaton DH (1992). Reversed vein graft stenosis: early diagnosis and management. J Vasc Surg.

